# Regulation of the Hsp104 Middle Domain Activity Is Critical for Yeast Prion Propagation

**DOI:** 10.1371/journal.pone.0087521

**Published:** 2014-01-23

**Authors:** Jennifer E. Dulle, Kevin C. Stein, Heather L. True

**Affiliations:** Department of Cell Biology and Physiology, Washington University School of Medicine, St. Louis, Missouri, United States of America; Van Andel Institute, United States of America

## Abstract

Molecular chaperones play a significant role in preventing protein misfolding and aggregation. Indeed, some protein conformational disorders have been linked to changes in the chaperone network. Curiously, in yeast, chaperones also play a role in promoting prion maintenance and propagation. While many amyloidogenic proteins are associated with disease in mammals, yeast prion proteins, and their ability to undergo conformational conversion into a prion state, are proposed to play a functional role in yeast biology. The chaperone Hsp104, a AAA+ ATPase, is essential for yeast prion propagation. Hsp104 fragments large prion aggregates to generate a population of smaller oligomers that can more readily convert soluble monomer and be transmitted to daughter cells. Here, we show that the middle (M) domain of Hsp104, and its mobility, plays an integral part in prion propagation. We generated and characterized mutations in the M-domain of Hsp104 that are predicted to stabilize either a repressed or de-repressed conformation of the M-domain (by analogy to ClpB in bacteria). We show that the predicted stabilization of the repressed conformation inhibits general chaperone activity. Mutation to the de-repressed conformation, however, has differential effects on ATP hydrolysis and disaggregation, suggesting that the M-domain is involved in coupling these two activities. Interestingly, we show that changes in the M-domain differentially affect the propagation of different variants of the [*PSI*+] and [*RNQ*+] prions, which indicates that some prion variants are more sensitive to changes in the M-domain mobility than others. Thus, we provide evidence that regulation of the M-domain of Hsp104 is critical for efficient prion propagation. This shows the importance of elucidating the function of the M-domain in order to understand the role of Hsp104 in the propagation of different prions and prion variants.

## Introduction

Protein aggregates pose a considerable challenge to cellular homeostasis and contribute to the pathogenesis of numerous neurodegenerative diseases. As such, protein misfolding and aggregation are guarded against by molecular chaperones, which act as the cell's first line of defense by maintaining proteostasis. In bacteria, fungi, and plants, the Hsp100 chaperones, together with Hsp70 and Hsp40 co-chaperones, are responsible for disaggregating protein aggregates and promoting cell survival and recovery from cell stress [1], [2]. The AAA+ ATPase Hsp104 is the primary disaggregase in the budding yeast *Saccharomyces cerevisiae* [3], [4]. Like its bacterial homolog, ClpB, the hexameric Hsp104 chaperone unwinds aggregates and threads protein substrates through a central channel to be refolded by Hsp70 chaperones [5], [6]. This function of Hsp104 is essential for cell survival post-heat stress (thermotolerance) as well as recovery from various other stresses [4], [7].

In addition to its role in protein disaggregation, Hsp104 is required for yeast prion propagation [8], [9]. Prions in yeast are self-replicating, cytoplasmically inherited protein aggregates that are proposed to have a functional role in the cell [10], [11], [12], [13], [14]. Prions are amyloid-like, consisting of cross-β sheet structures that are highly stable and resistant to high temperature and detergents [15]. Prion propagation and maintenance in yeast requires efficient fragmentation to generate prion-competent oligomers, or propagons, which can be transmitted to daughter cells. Hsp104 is proposed to remodel large prion aggregates to produce propagons, thereby generating more “free ends” that are required for additional monomer templating [16], [17], [18], [19]. Furthermore, Hsp104 has been implicated in the selection of prion variants, which are conformationally distinct aggregates of the same protein sequence that are akin to mammalian prion strains [20]. Alterations in the stability of different prion variant structures are proposed to govern such selection by influencing the interaction and fragmentation of prion aggregates by Hsp104 [20], [21], [22].

One of the best-characterized yeast prions is formed from the reversible aggregation of the translation termination factor, Sup35 [23], [24], [25]. This prion, called [*PSI*+], has been proposed to play a role in creating genetic diversity by promoting translation of normally silent regions of the genome [11], [26]. Interestingly, the *de novo* formation of [*PSI*+] is regulated by another yeast prion, [*RNQ*+], resulting from the aggregation of the Rnq1 protein [27], [28], [29], [30], [31]. The formation of [*PSI*+] has been shown to increase cell viability under various stresses, but can be detrimental in others, suggesting that the formation and propagation of [*PSI*+] is an important biological process that must be strictly regulated, in part by Hsp104 [11], [32], [33], [34], [35].

Hsp104 can be divided into five functionally distinct, yet cooperative, domains. The N-terminal domain is not required for either prion propagation or thermotolerance, but has been proposed to be a site for substrate binding, as well as an interaction site for the Hsp70 and Hsp40 co-chaperones [36], [37]. Two nucleotide-binding domains, NBD1 and NBD2, bind and hydrolyze ATP to stabilize hexamer formation and catalyze the disaggregation of substrates [38]. The role of the C-terminal domain is still not well understood, as it is unnecessary for prion propagation and thermotolerance, yet both activities are affected by mutations in this domain [36], [39], [40], [41]. Finally, the linker region, or middle domain (M-domain), is proposed to regulate both ATP hydrolysis and substrate disaggregation by coordinating the actions of NBD1 and NBD2 [36], [42], [43].

The M-domain is a coiled-coil insertion between NBD1 and NBD2 and is characteristic of Hsp100 chaperones that function as disaggregases, including the bacterial homolog, ClpB [44], [45]. In both Hsp104 and ClpB, the M-domain regulates ATP hydrolysis [46], [47], [48], is essential for substrate disaggregation [49], [50], [51], and mediates the interaction with Hsp70 chaperones [49], [52], [53], [54]. Biochemical, genetic, and structural studies with both Hsp104 and ClpB suggest that the M-domain projects from the body of the hexamer and makes contact with the NBD1 of neighboring subunits [43], [46], [48], [55], [56]. Recent data suggest that the M-domain of ClpB can occupy two distinct functional states, repressed and de-repressed [48]. In the repressed state, the M-domain is nestled against the body of the hexamer, maintaining contact with a neighboring NBD1. Interaction with Hsp70 is proposed to promote a shift of the M-domain away from NBD1 to the de-repressed conformation, thereby increasing the ATPase activity and, in turn, promoting substrate disaggregation [43], [48]. ClpB mutations that stabilize the M-domain in the repressed state prevent substrate-stimulated ATPase activity and decrease substrate disaggregation [48]. On the other hand, mutations in ClpB that stabilize a de-repressed state of the M-domain result in hyperactivity and cause toxicity in vivo [43], [48]. Thus, the mobility of the M-domain plays a significant role in regulating the activity of ClpB. As such, elucidating the function of the M-domain in regulating Hsp104 activity is critical to understanding how Hsp104 is able to disaggregate a broad range of substrates.

In the present study, we generated mutations in the M-domain of Hsp104 analogous to the previously characterized repressed and de-repressed mutations in ClpB [48], [54] and investigated their effect on Hsp104 activity and yeast prion propagation. We found that an M-domain mutation predicted to repress the mobility of the M-domain prevented thermotolerance and prion propagation. Strikingly, mutations that we hypothesized would de-repress Hsp104 M-domain function also resulted in prion elimination, but in a prion variant-specific manner. Our data show that the mobility of the M-domain regulates Hsp104 disaggregase activity and suggest that changes in this mobility have significant consequences for processing different substrates.

## Materials and Methods

### Strain and Plasmid Construction

All *S. cerevisiae* strains were derivatives of 74-D694 and were grown using standard culture techniques. Strains were grown in YPD (1% yeast extract, 2% peptone, 2% glucose) or synthetic defined media (0.67% yeast nitrogen base, 2% glucose) lacking amino acids that correlated with plasmid auxotrophic markers.

For expression of the Hsp104 mutants in vivo, point mutations in *HSP104* were generated by bridge PCR using as the template, pRS313-phs-*HSP104* [5] (kindly provided by B. Bukau), which expresses *HSP104* from the *HSP104* promoter (phs). Bridge PCR products and pRS313-phs-*HSP104* were digested with EcoRI and Bsu36I, which are endogenous restriction sites in the *HSP104* open-reading frame, and ligated together. Hsp104 mutants were also cloned into *pProEx-HTb-HSP104* [40] (kindly provided by J. Glover) by the same digestion and ligation. The pRS313-phs-*hsp104-V426I* plasmid was generated by PCR amplifying genomic DNA from the EMS mutagenized strain, then digesting and ligating as described.

The strong and weak variants of [*PSI*+] in 74-D694 were previously characterized and kindly provided by Y. Chernoff and S. Liebman [8], [57]. To generate strains propagating each of the [*PSI*+] variants and harboring the Hsp104 mutants, cells propagating each variant were mated to an *hsp104Δ* (*hsp104::leu2*) strain and diploids were selected. The mutant pRS313-phs-*HSP104* plasmids were transformed into the heterozygous diploids, the diploids were sporulated, and haploids were selected on media lacking histidine and leucine. Colonies were verified as haploids by mating-type testing.

The [*RNQ*+] variant yeast strains [58] were kindly provided by the Liebman lab. To create strains carrying both the mutant Hsp104 plasmids and the [*RNQ*+] variants, we created *HSP104* plasmid shuffle strains. First, pRS316-phs-*HSP104* [36] (kindly provided by J. Weissman) was first transformed into cells propagating each of the [*RNQ*+] variants. *HSP104* on the chromosome was deleted by transforming the *hphMX4* cassette amplified from pAG32 using oligonucleotides 5′GAAAAAAGAAATCAACTACACGTACCATAAAATATACAGAATATCAGCTGAAGCTTCGTACGC and 5′GATTCTTGTTCGAAAGTTTTTAAAAATCACACTATATTAAAGCATAGGCCACTAGTGGATCTG, containing flanking homology to the *HSP104* promoter and terminator. Deletion of *HSP104* was confirmed by PCR in Ura+ HygB^R^ colonies. These strains were then transformed with each of the mutant pRS313-phs-*hsp104* plasmids, selected on media lacking histidine and uracil, grown overnight in liquid media lacking just histidine, and then plated on media lacking histidine and containing 5-fluoroorotic acid (US Biologicals) to select for cells that had lost the pRS316-phs-*HSP104* plasmid. Colonies that were His+ ura− were used for further analysis.

### EMS mutagenesis screen

The strong [*PSI+*] yeast strain was subjected to EMS mutagenesis as previously described [59]. Two cultures with viabilities of about 17% were plated to determine changes in color. Candidates were selected based on color phenotype and were initially identified as mutations in *HSP104* by back-crossing to an *hsp104Δ* strain and analyzing the progeny for segregation of the prion-dependent nonsense suppression phenotype. Genomic DNA was PCR amplified and sequenced to identify the point mutations in *HSP104*.

### Fluorescence Microscopy

Cells expressing pRS316*CUP1*-*SUP35NM-GFP* [60] (kindly provided by S. Liebman) were grown in media containing 50 µM CuSO_4_ for four hours to induce expression of *SUP35NM-GFP*. Cells expressing Sup35NM-GFP were imaged in water at room temperature on an Olympus Bmax-60F microscope containing a 1.35NA 100X UPlanApo objective lens, spinning disc Confocal Scanner Unit (CSU10). Images were captured using a Stanford Photonics XR-Mega10 ICCD camera with QED software and analyzed by ImageJ.

### SDD-AGE Analysis

Cells were lysed by disruption of the membranes with glass beads in Sup35 PEB buffer (25 mM Tris-HCl pH 7.5, 50 mM KCl, 10 mM MgCl_2_, 1 mM EDTA, 10% Glycerol, mini EDTA-free protease inhibitors (Roche), Aprotinen (Sigma) and PMSF (Sigma)) or Rnq1 PEB buffer (25 mM Tris-HCl pH 7.5, 100 mM NaCl, 1 mM EDTA, mini EDTA-free protease inhibitors, 0.5 mM DTT, 3 mM PMSF, 5 µg/mL pepstatin, and 40 mM NEM). Samples were incubated in sample buffer at room temperature for seven minutes, then separated on a 1.5% agarose gel. The protein distribution was analyzed by western blot with anti-Sup35 or anti-Rnq1 antibodies.

### Hsp104 Purification

Recombinant Hsp104 was expressed and purified from *E. coli* cells as previously described [61]. After purification, the pool of recombinant Hsp104 was separated on an S-300 gel filtration column to isolate Hsp104 monomers. Purified, monomeric Hsp104 was concentrated and frozen at -80°C in storage buffer (20 mM Tris pH 8.0, 100 mM NaCl, 10 mM MgCl_2_, 2 mM EDTA, 10% glycerol).

### ATP Hydrolysis Assays

The Malachite green assay was used to measure the rates of ATP hydrolysis [38]. Purified protein (2 µg) was incubated with 5 mM ATP in buffer (40 mM Tris-HCl pH 7.5, 175 mM NaCl, 5 mM MgCl_2_, 0.02% Triton X-100) at 37°C. At each minute over a time course of 12 minutes, Malachite green dye was added to the sample and the reaction stopped by the addition of 34% citric acid. The absorbance was measured at 650 nm and the concentration of free phosphate was calculated based on a standard of KH_2_PO_4_ and normalized to the sample containing no Hsp104.

### Glycerol Gradients

Purified Hsp104 (50 µg) was incubated with 5 mM ATP in buffer (40 mM Tris-HCl pH 7.5, 175 mM NaCl, 5 mM MgCl_2_, 0.02% Triton X-100), then centrifuged at 34 k rpm for 18 hours through a 4 mL linear (10–35%) glycerol gradient containing 5 mM ATP. The gradients were fractionated and equal volumes of each fraction were analyzed by SDS-PAGE and western blot using an anti-Hsp104 antibody. Individual bands from each fraction were quantified using ImageJ and reported as a percent of total Hsp104.

### Thermotolerance

An equal number of *hsp104*Δ cells maintaining plasmids that expressed *HSP104*, *hsp104-V426I, hsp104-V426C*, *hsp104-K480C*, *hsp104-Y507D*, *hsp104-D434A*, or an empty vector control, were treated at 37°C in equal volumes for 30 minutes to induce *HSP104* expression, then heat-shocked at 50°C. At 10, 15, 20, 25, and 30 minutes during heat shock, samples were taken and spotted on media lacking histidine in a five-fold dilution.

### Luciferase Refolding

An *hsp104*Δ strain containing plasmids expressing *HSP104*, *hsp104-V426I, hsp104-V426C*, *hsp104-K480C*, *hsp104-Y507D*, *hsp104-D434A*, or an empty vector control, were transformed with pRS316-*GPD*-luciferase [5] (kindly provided by B.Bukau). Cells were grown at 37°C for one hour, then heat-shocked at 44°C for one hour. Fifty minutes into the heat shock, cycloheximide (Sigma) was added to the culture to block protein synthesis. At various times during recovery at 30°C, 100 µl samples were taken and 50 µl of 1 mM beetle luciferin (Promega) was added. Luminescence was measured on a Sirius luminometer. The resolubilization of luciferase was calculated by dividing the measured luminescence at each time point by the measured luminescence prior to heat shock and normalized to the luminescence measured immediately after heat shock.

## Results

### Hsp104 M-domain mutant, *hsp104-V426I*, causes sectoring [*PSI*+] phenotype

**Figure 1:**
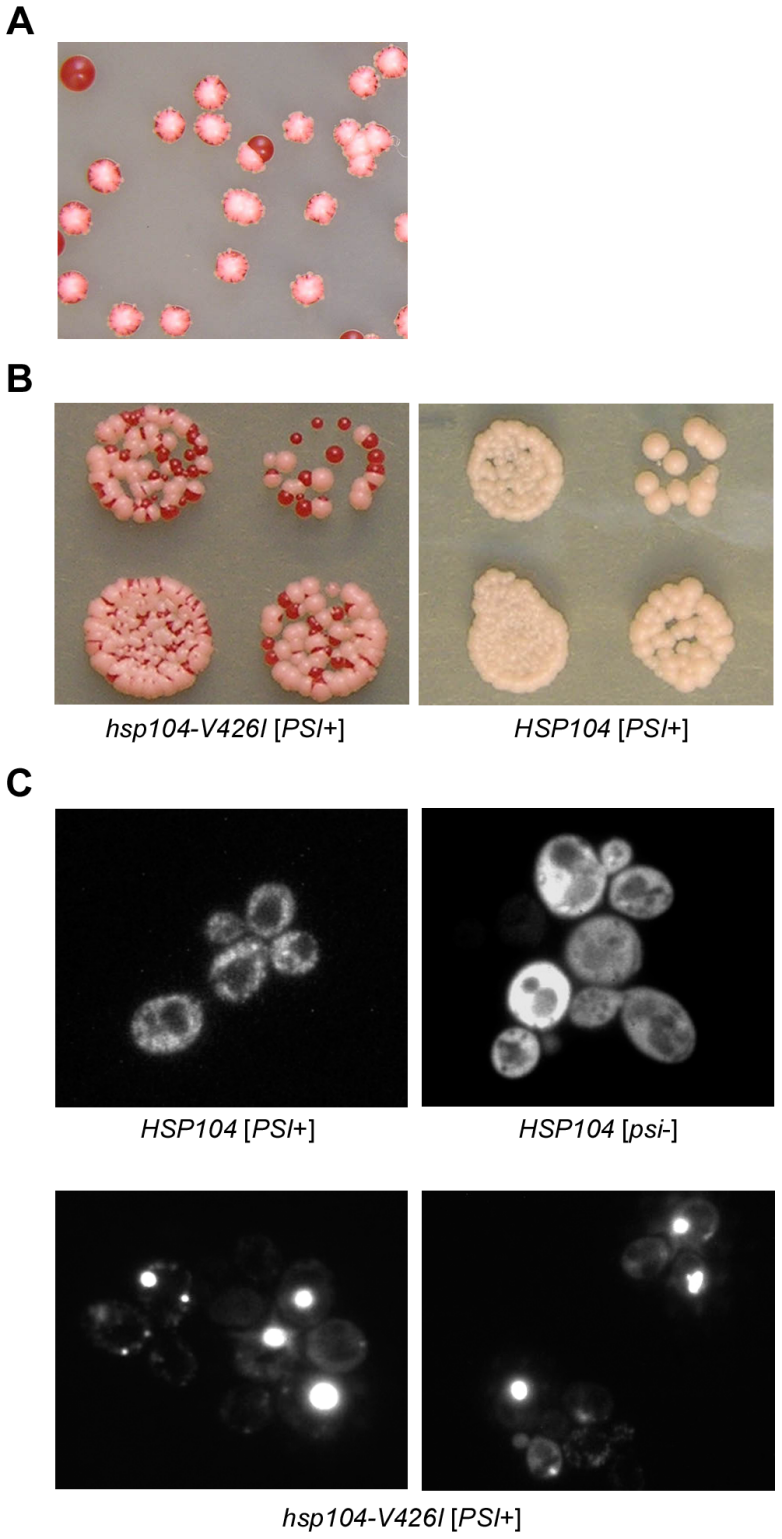
A point mutation in Hsp104 destabilizes [*PSI*+]. (A–B) Cells containing *hsp104-V426I* or *HSP104* were plated onto solid rich medium (YPD) to illustrate the destabilizing effect that this mutation has on the [*PSI*+] phenotype. (A) In the presence of *hsp104-V426I*, [*PSI*+] is lost in a fraction of the buds, generating sectors of [*psi*−] cells (phenotypically red) in the [*PSI*+] colony. (B) Cells expressing *hsp104-V426I* lose the [*PSI*+] prion more frequently than *HSP104* cells. (C) The copper-inducible fluorescent protein, Sup35NM-GFP, was ectopically expressed in *hsp104-V426I* [*PSI*+] cells along with wild type [*PSI*+] and [*psi*−] cells. Fluorescence imaging was performed on an Olympus confocal microscope and representative images are shown.

We performed a genetic screen to identify factors important for aggregation of the translation termination factor Sup35 and the resulting propagation of the [*PSI*+] prion. To identify candidates, we used a color-based phenotypic assay established to follow [*PSI*+] propagation. In this assay, a premature termination codon is present in the *ADE1* gene, in the *ade1-14* allele, which prevents completion of the adenine biosynthesis pathway. Disruption of adenine biosynthesis at this point in the pathway causes the accumulation of a red-pigmented intermediate and prevents cells from growing on media lacking adenine. Translational read through of the premature termination codon in *ade1-14* leads to completion of the pathway, resulting in cells that are phenotypically light pink or white when grown on rich media (YPD) and are able to grow on media lacking adenine. When Sup35 is not aggregated and maintains its normal function (in non-prion-containing [*psi*−] cells), translation termination is efficient, and the *ade1-14* colonies appear red in color and do not grow on media lacking adenine. Conversely, when Sup35 is in a prion state, it is aggregated and less functional, and the [*PSI*+] colonies are Ade+ (light pink in color on YPD and able to grow on media lacking adenine). From our screen, we identified a candidate that caused the [*PSI+*] cells to change from a light pink phenotype to a sectoring colony color phenotype (Figure 1A). This indicates that a fraction of the cells in a colony did not inherit [*PSI*+] propagons, causing those cells to become [*psi*−] and phenotypically red. All of the progeny from those [*psi*−] cells will also be [*psi*−] and this results in a sectoring colony color phenotype. Moreover, this candidate caused a corresponding increase in the mitotic loss of the [*PSI*+] prion (all red [*psi*−] colonies) as compared to wild type *HSP104* cells in which loss of [*PSI*+] is rarely observed (Figure 1B and data not shown). By genetic testing, we discovered that this phenotype resulted from a point mutation in Hsp104. We sequenced *hsp104* in this strain and identified the mutation as *hsp104-V426I*. To confirm that the [*PSI*+] inheritance defect resulted from this mutation, we made the *hsp104-V426I* mutation in an unmutagenized [*PSI*+] strain and used this strain for all further analyses.

To determine whether this Hsp104 mutant was affecting the aggregation of Sup35 in [*PSI*+] cells, we transformed the *hsp104-V426I* mutant strain with a plasmid expressing *SUP35NM-GFP* and analyzed the Sup35 aggregation pattern by fluorescence imaging. In *hsp104-V426I* samples, we observed cells that contained fluorescent foci indicative of Sup35 aggregates, as well as cells that displayed diffuse fluorescence similar to [*psi*−] cells (Figure 1C). Interestingly, the *hsp104-V426I* cells with fluorescent foci contained a single or a few large fluorescent foci, unlike the wild type [*PSI+*] cells, which contained multiple, small fluorescent foci (Figure 1C). Thus, we conclude that the mutant *hsp104-V426I* affects the aggregated state of the prion determinant Sup35, thereby altering the [*PSI+*] phenotype.

### Hsp104-V426 is located in the coiled-coil M-domain

To determine how this mutation may be affecting Hsp104 function, we examined the structural models of Hsp104 to identify where this residue is located [55], [56]. We discovered that V426 appears to be located in the first helix of motif 1 of the M-domain and is analogous to the L424 residue in ClpB. Recently, functional analysis of the M-domain of ClpB suggested that the L424 residue helps mediate the mobility and position of the coiled-coil M-domain by contributing to the interaction between the M-domain and the NBD1 of the neighboring subunit [48]. Another residue in the M-domain of ClpB, Y503, was also shown to regulate M-domain mobility through an interaction with NBD1 [48]. The ClpB-Y503D mutation led to a pronounced decrease in KJE-dependent (DnaK-DnaJ-GrpE) ClpB disaggregation activity [47]. More recently, ClpB-Y503D was shown to increase the rate of substrate-stimulated ATP hydrolysis and cause toxicity when expressed in bacteria grown at high temperatures [48]. The Y503D mutation in ClpB was proposed to stabilize a de-repressed conformation of the M-domain, in which there is a constitutive loss of contact of the M-domain with NBD1, thereby causing ClpB hyperactivity. We hypothesized that the Hsp104-V426I mutation that we identified in our screen might disrupt the mobility of the Hsp104 M-domain to alter prion propagation.

We set out to further assess the role that mobility of the M-domain has on the function of Hsp104 as compared to Hsp104-V426I. Mutations in the ClpB M-domain have been classified as repressed or de-repressed, which have contrasting effects on the function of ClpB [48], [54]. A recent study analyzed how these two classes of mutants modulated ClpB ATPase activity, disaggregation activity, and cell growth [48]. We created analogous mutations in the M-domain of Hsp104 to determine if the effects of these mutants on disaggregase function are conserved between the chaperones. This included the putative repressed Hsp104-D434A mutation (homologous to ClpB-E432A), along with Hsp104-K480C and Hsp104-Y507D, which are homologous to the de-repressed mutations of ClpB-K476C and ClpB-Y503D, respectively. We also generated Hsp104-V426C that is analogous to the ClpB-L424C mutation that was used to characterize the interaction of the M-domain with NBD1 [48]. We first analyzed the biochemical properties and disaggregation activities of the Hsp104 mutants to determine if they display similar functional effects as their counterparts in ClpB. Then, we analyzed the effect of these mutants on the propagation of two yeast prions - [*PSI*+] and [*RNQ*+].

### M-domain mutants display varying levels of ATPase activity and hexamer formation

**Figure 2:**
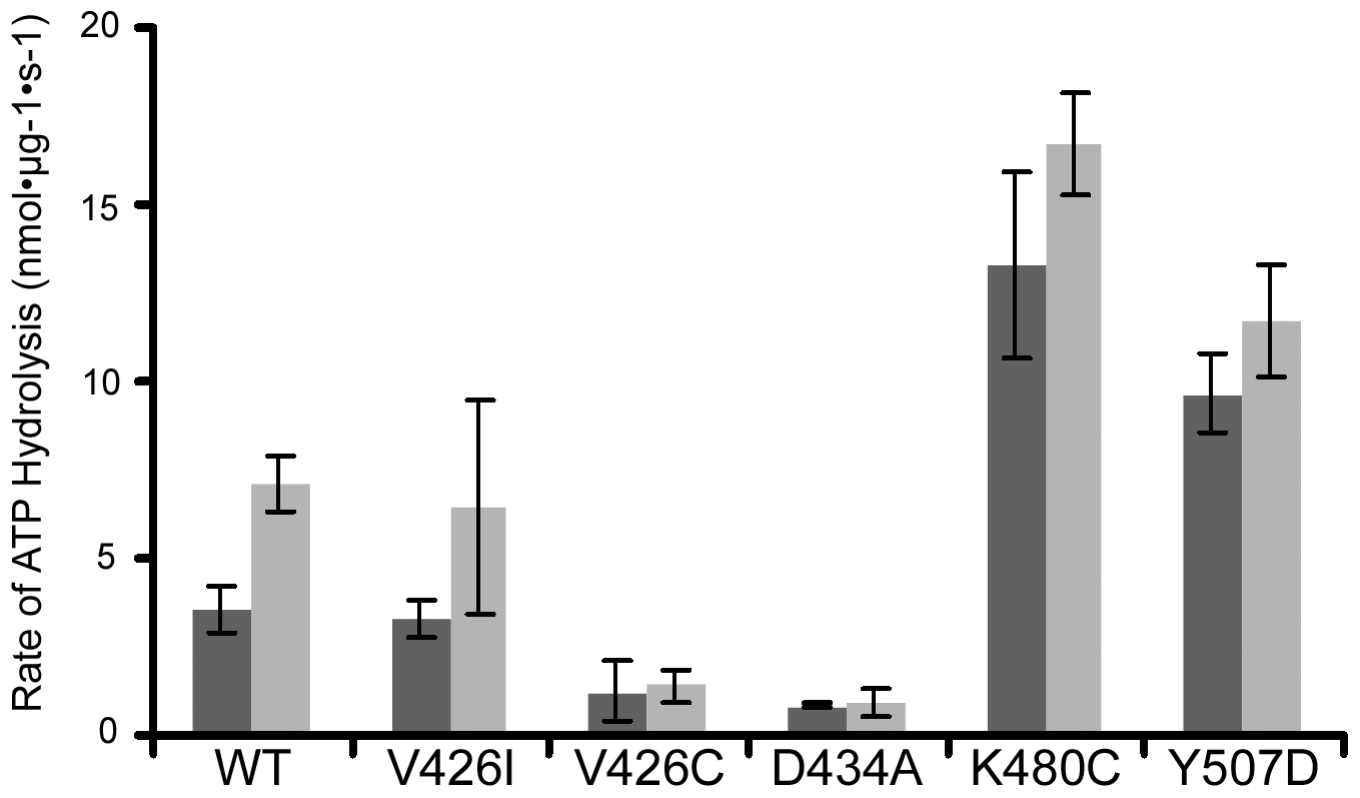
Hsp104 M-domain mutants affect ATPase activity. The ATPase activity of recombinant wild type (WT) Hsp104, Hsp104-V426I, Hsp104-V426C, Hsp104-D434A, Hsp104-K480C, and Hsp104-Y507D was measured by the Malachite Green assay after incubation of 2 µg of protein with 5 mM ATP at 37°C either in the absence (black) or presence (grey) of 0.25 mg/mL β-casein. The amount of free inorganic phosphate in each sample was calculated from analysis of phosphate standards. For each protein, the average initial rate of ATP hydrolysis is plotted. Each protein was assayed in quadruplicate from two separate purification preparations and the error bars reflect standard deviation between the samples.

The M-domain regulates ATPase activity by interacting with the NBD1 of the neighboring subunit in the hexamer and coordinating ATP binding and hydrolysis between NBD1 and NBD2 [46], [47], [62]. Both the repressed and de-repressed ClpB mutants showed basal levels of ATP hydrolysis similar to wild type ClpB [48]. However, the de-repressed ClpB mutants had significantly higher substrate-stimulated ATPase activity [48]. To determine if the analogous M-domain mutants in Hsp104 had a similar impact on ATPase activity, we purified recombinant wild type Hsp104 and the M-domain mutants and measured both the basal and substrate-stimulated ATP hydrolysis rates by the Malachite Green assay [38]. Interestingly, Hsp104-V426I, the mutant identified in our screen that altered [*PSI*+] propagation, maintained wild type rates of both basal and substrate-stimulated ATP hydrolysis (Figure 2). By contrast, Hsp104-D434A and Hsp104-V426C exhibited decreased basal levels of ATPase activity as compared to wild type, while Hsp104-K480C and Hsp104-Y507D displayed higher rates of basal ATPase activity (Figure 2). Additionally, wild type Hsp104, Hsp104-V426I, Hsp104-K480C, and Hsp104-Y507D all exhibited increased rates of ATP hydrolysis in the presence of substrate (Figure 2). However, addition of substrate did not increase the ATP hydrolysis rate above the basal level for Hsp104-D434A or Hsp104-V426C.

**Figure 3:**
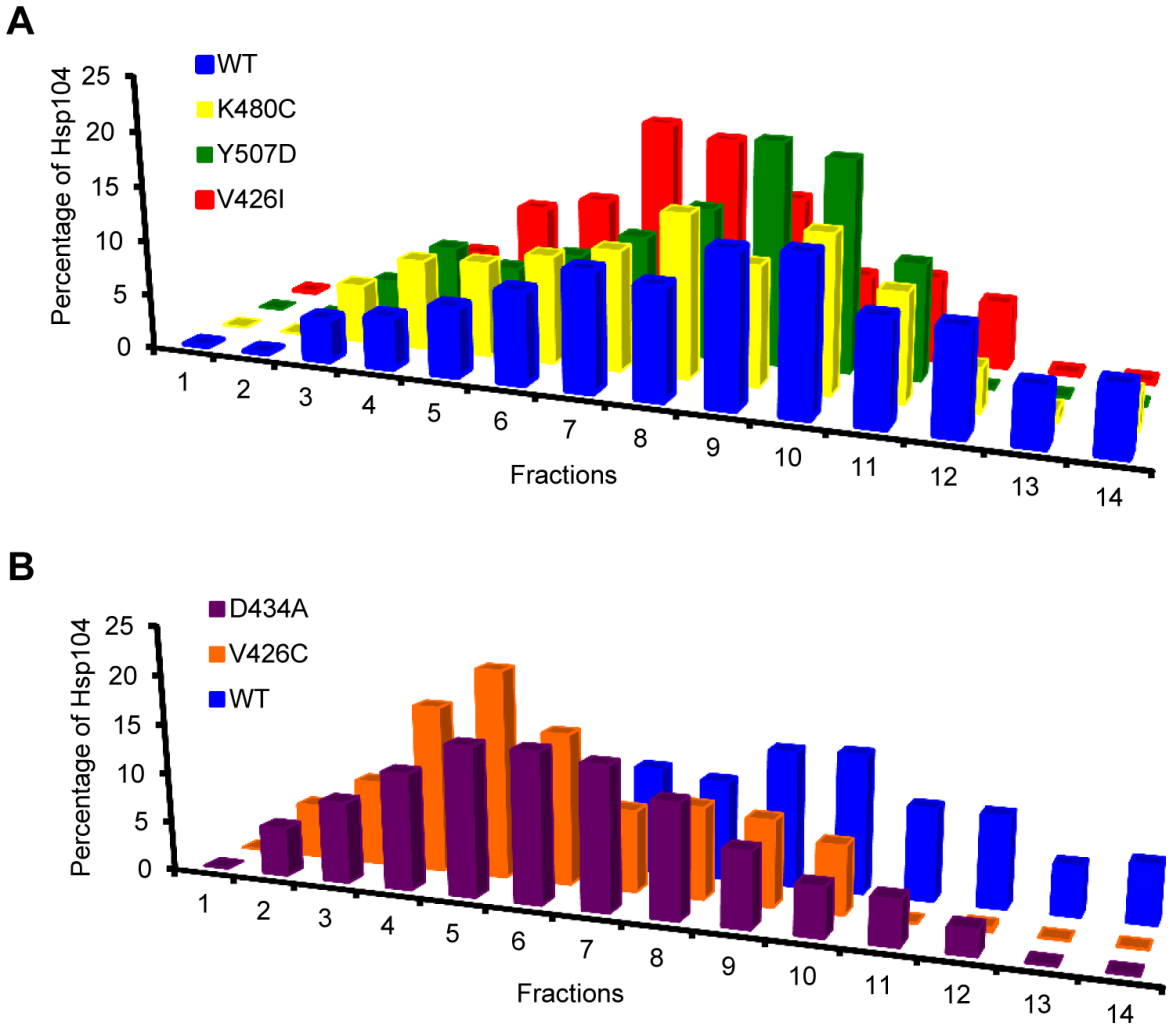
The M-domain plays a role in hexamer formation. The oligomeric distribution of recombinant wild type (WT) Hsp104 (blue, A & B) and (A) Hsp104-V426I (red), Hsp104-K480C (yellow), and Hsp104-Y507D (green), or (B) Hsp104-V426C (orange) and Hsp104-D434A (purple), was analyzed by ultracentrifugation through a linear glycerol gradient in the presence of 5 mM ATP. Equal fractions from the gradients were collected and analyzed by western blot with an anti-Hsp104 antibody. The amount of Hsp104 in each fraction was quantified by ImageJ and graphed as a fraction of the total Hsp104. The gradients were repeated twice with recombinant protein from two separate recombinant protein purification preparations.

The ATPase activity of Hsp104 depends on the hexameric state of the chaperone. Hsp104 mutants that inhibit hexamer formation also inhibit ATP hydrolysis [38]. In addition to regulating ATPase activity, the M-domain has also been implicated in hexamer formation and stability [46]. We reasoned that the decreased rates of ATP hydrolysis that we observed for a subset of the M-domain mutants might correlate with inefficient hexamer formation or a change in stability of the hexameric state. To test this, we incubated the purified Hsp104 M-domain mutants with ATP and then subjected the samples to ultracentrifugation on a linear glycerol gradient. Hsp104-V426I, Hsp104-K480C, and Hsp104-Y507D all formed hexamers and separated on the gradient like wild type Hsp104 (Figure 3A). Alternatively, Hsp104-D434A and Hsp104-V426C, which displayed decreased rates of ATP hydrolysis, also displayed a decrease in stable hexamer formation (Figure 3B). Thus, the apparent lack of efficient hexamer formation of Hsp104-D434A and Hsp104-V426C likely contributes to the observed decrease in their ATPase activity. This suggests that the Hsp104-D434A and Hsp104-V426C mutations decrease the ability of the M-domain to regulate ATPase activity and hexamer formation, presumably by stabilizing a repressed conformation. On the other hand, Hsp104-K480C and Hsp104-Y507D appear to cause hyperactivity, resulting in increased basal ATPase activity and an apparent de-repressed state.

### Hsp104-K480C and Hsp104-Y507D cause cellular toxicity in a temperature-dependent manner

**Figure 4:**
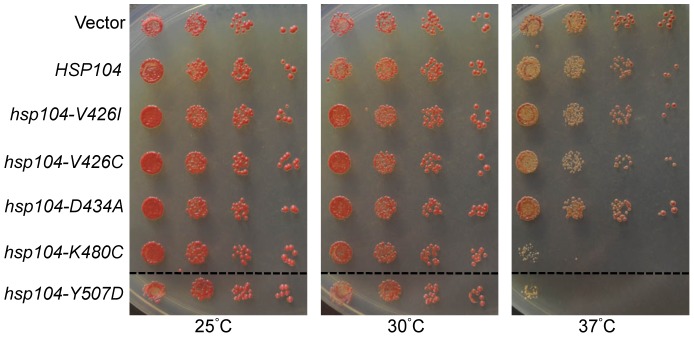
Hsp104-K480C and Hsp104-Y507D are toxic at high temperatures. *hsp104*Δ strains expressing wild type *HSP104*, *hsp104-V426I*, *hsp104-V426C*, *hsp104-D434A*, *hsp104-K480C*, or *hsp104-Y507D* from a *HIS3*-containing plasmid, were plated on solid medium lacking histidine and grown at 25, 30, or 37°C to assess temperature-dependent growth defects, as compared to an empty vector control (Vector). Dashed lines represent different parts of the same plate that have been cropped for clarity. These spottings are representative of three independent experiments.

Since the repressed and de-repressed ClpB mutants showed a difference in cell viability at high temperatures [48], we next tested whether any of the Hsp104 M-domain mutants showed temperature-dependent growth defects. We created *hsp104Δ* strains that maintained a plasmid expressing the Hsp104 mutant (or a wild type control) from its native promoter and as the only copy of Hsp104 (Figure S1). We grew these strains on media that selected for the plasmid at 25, 30, and 37°C. At 25 and 30°C, all the mutant strains grew similar to wild type *HSP104* cells (Figure 4). At 37°C, however, both *hsp104-K480C* and *hsp104-Y507D* strains were unable to grow (Figure 4). This heat sensitive growth defect is similar to that of the analogous ClpB mutants, ClpB-K476C and ClpB-Y503D, which were shown to be hyperactive mutants that resulted in cellular toxicity [48], [54]. For comparison, a vector-only control was also plated, and this strain shows normal cell growth. Therefore, the toxicity associated with these Hsp104 mutations is not due to a lack of Hsp104 or a simple loss-of-function, but suggests a toxic gain-of-function of these mutants that impairs cell growth. As this toxicity is observed at a temperature that induces more Hsp104 expression (37°C), it is possible that constitutive expression of these two mutants is detrimental to cellular homeostasis and decreases cell viability due to an enhanced interaction with a natural, essential substrate.

### M-domain mutants present varying levels of thermotolerance and non-prion aggregate disaggregation

**Figure 5:**
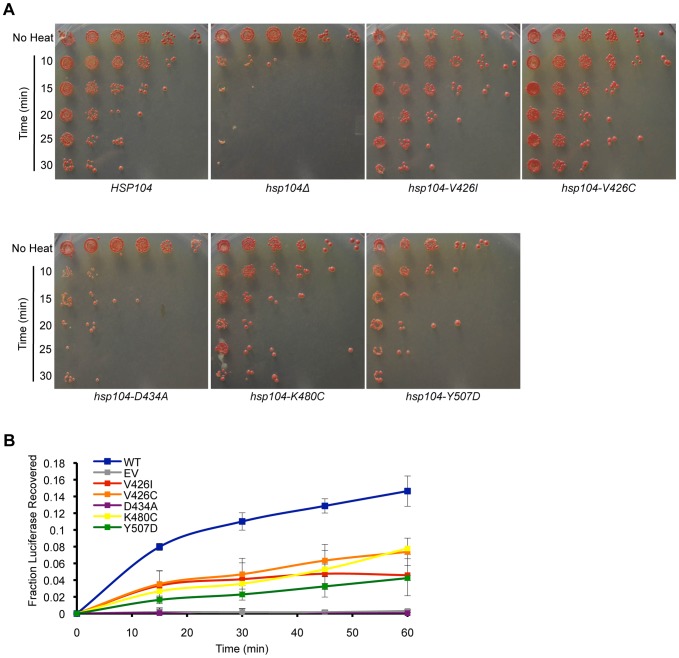
M-domain mutants have differing effects on the ability to disaggregate non-prion substrates. (A) *hsp104*Δ strains expressing wild type *HSP104*, *hsp104-V426I*, *hsp104-V426C*, *hsp104-D434A*, *hsp104-K480C*, *hsp104-Y507D* from a *HIS3*-containing plasmid, or an empty vector control (*hsp104*Δ), were heat shocked to measure the mutants' ability to confer thermotolerance. Cultures were grown at 37°C to induce Hsp104 expression, then heat shocked at 50°C for various amounts of time, as compared to controls with no heat shock (No Heat), serially diluted five-fold, and spotted on medium lacking histidine to assess viability. Data are representative of three individual experiments. (B) *hsp104*Δ strains containing a plasmid expressing luciferase and expressing wild type (WT) *HSP104* (blue), *hsp104-V426I* (red), *hsp104-V426C* (orange), *hsp104-D434A* (purple), *hsp104-K480C* (yellow), *hsp104-Y507D* (green), or an empty vector (EV) control (gray) were grown at 37°C to induce Hsp104 expression, then heat shocked at 44°C for an hour to induce luciferase aggregation. At the indicated times during recovery at 30°C, samples were taken, luciferin was added, and the luminescence was measured. The graph represents the amount of luciferase recovered as a fraction of the total luciferase before heat shock. Three separate samples for each mutant were analyzed and error bars reflect standard deviation between the samples.

Hsp104 is required for cell viability following heat shock (thermotolerance) [7]. To confer thermotolerance, Hsp104 must disaggregate non-prion substrates that aggregate as a consequence of the heat stress. The M-domain of Hsp104 (and ClpB) is proposed to affect the disaggregation of substrates by providing a site for an interaction with co-chaperones (Hsp70 and Hsp40 in yeast, DnaK and DnaJ in bacteria) [47], [52]. Thus, mutations in the M-domain, which abrogate the interaction with co-chaperones, have a negative effect on the ability of Hsp104 to disaggregate substrates [53], [63]. Furthermore, as the ATPase activity and disaggregation activity are interdependent, mutations in the M-domain that affect the regulation of ATPase activity may also affect the disaggregation mechanism. Therefore, we investigated the general disaggregation activity of the Hsp104 M-domain mutants in vivo by analyzing their ability to confer thermotolerance to yeast. As above, we transformed an *hsp104*Δ strain with a plasmid expressing each of the M-domain mutants from the native promoter, wild type *HSP104*, or an empty vector control. We then grew these strains to mid-logarithmic phase at 30°C, moved them to 37°C to induce expression of Hsp104, then heat shocked the strains at 50°C for various amounts of time before plating the cells to assess viability. We found that, like the *hsp104*Δ strain, *hsp104-D434A* cells were not thermotolerant (Figure 5A). Conversely, both *hsp104-V426I* and *hsp104-V426C* cells maintained wild type thermotolerance (Figure 5A). Interestingly, the two mutants with the highest ATPase activity, *hsp104-K480C* and *hsp104-Y507D*, presented an intermediate level of thermotolerance, where the amount of cell recovery after heat stress was between that of wild type *HSP104* and *hsp104*Δ strains (Figure 5A). This loss of thermotolerance, however, is likely due to the temperature-dependent cytotoxicity associated with *hsp104-K480C* and *hsp104-Y507D* cells (Figure 4).

We next tested the ability of the M-domain mutants to disaggregate heat-aggregated luciferase, which has previously been shown to be a substrate of Hsp104 [36]. The strains described above, each containing a plasmid expressing either wild type or mutant Hsp104, were transformed with a plasmid expressing luciferase. After growing to mid-logarithmic phase, these strains were grown for an hour at 37°C to induce Hsp104 expression and were then heat shocked for an hour at 44°C to induce luciferase aggregation. After heat shock, the cells were allowed to recover at 30°C and we took samples over time and quantified the relative amount of luminescence, which represents the amount of luciferase resolubilized and refolded. As we saw in the thermotolerance assays, *hsp104-D434A* cells resembled the *hsp104*Δ strain harboring a vector only, in that there appeared to be no increase in the amount of resolubilized luciferase over time (Figure 5B). This indicates that Hsp104-D434A has a general defect in disaggregation. Cells expressing *hsp104-K480C* and *hsp104-Y507D*, on the other hand, showed luciferase recovery at rates that were about half of that observed in wild type *HSP104* cells. However, this may again be because these cells exhibit cytotoxicity at higher temperatures. Interestingly, Hsp104-V426I and Hsp104-V426C also showed a two-fold decrease in lucerifase refolding capability, despite being fully functional in conferring thermotolerance. This suggests that these mutations impair the ability of Hsp104 to disaggregate substrates, which agrees with the sectoring [*PSI*+] phenotype that we originally observed with Hsp104-V426I.

### Hsp104 M-domain mutants vary in their ability to propagate variants of the [*PSI*+] prion

**Figure 6:**
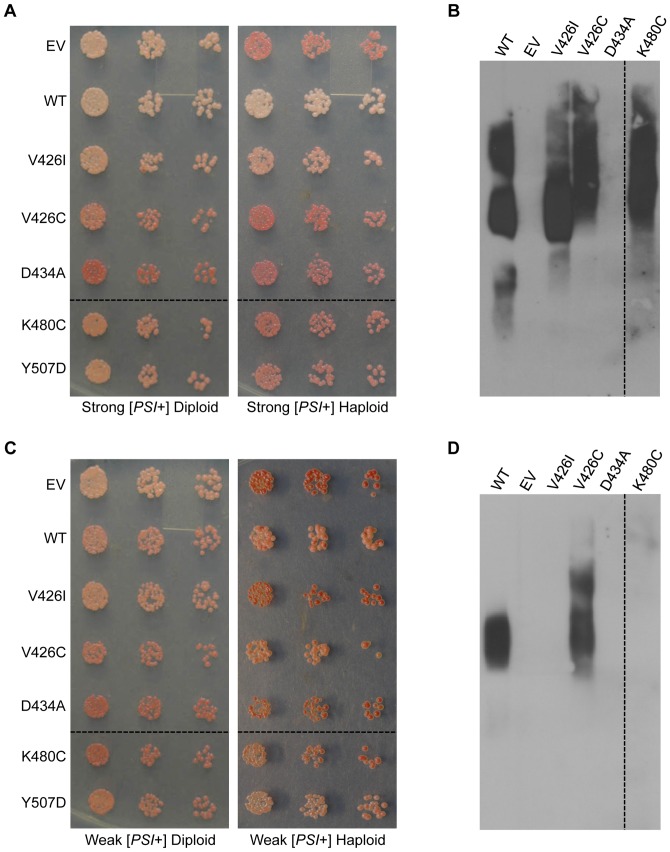
M-domain mutants differentially affect propagation of strong and weak variants of [*PSI*+]. (A) Heterozygous *HSP104*/*hsp104*Δ diploids or *hsp104*Δ haploids propagating strong [*PSI*+] and containing plasmids expressing *HSP104* (WT), *hsp104-V426I*, *hsp104-V426C*, *hsp104-D434A*, *hsp104-K480C*, *hsp104-Y507D*, or an empty vector control (EV), were normalized, serially diluted five-fold, and spotted on medium to select for the plasmid. Dashed lines represent different parts of the same plate that have been cropped for clarity. (B) Strong [*PSI*+] *hsp104*Δ haploids harboring the indicated Hsp104 plasmid or containing an empty vector control (EV) were subjected to SDD-AGE and western blot with an antibody against Sup35. The dashed line represents different parts of the same gel that have been cropped for clarity. This is one representative of three separate experiments. (C) Heterozygous *HSP104*/*hsp104*Δ diploids or *hsp104*Δ haploids propagating weak [*PSI*+] and containing plasmids expressing *HSP104* (WT), *hsp104-V426I*, *hsp104-V426C*, *hsp104-D434A*, *hsp104-K480C*, *hsp104-Y507D*, or an empty vector control (EV), were normalized, serially diluted five-fold, and spotted on medium selecting for the plasmid. Dashed lines represent different parts of the same plate that have been cropped for clarity. (D) The weak [*PSI*+] parental strain (WT) and weak [*PSI*+] haploids harboring the indicated Hsp104 plasmid or an empty vector control (EV) were subjected to SDD-AGE and western blot with an antibody against Sup35. The dashed line represents different parts of the same gel that have been cropped for clarity. This is one representative of five separate experiments.

Given the varying effects of the M-domain mutants on ATPase and disaggregase activity, we next sought to ascertain the effect of the M-domain mutants on [*PSI*+] propagation. We first demonstrated that Hsp104-V426I caused a defect in the propagation of one [*PSI*+] variant, strong [*PSI*+], and resulted in sectoring colonies (Figure 1A). To investigate the effect of the remaining M-domain mutants on strong [*PSI*+] propagation, we transformed a strong [*PSI*+] heterozygous *HSP104*/*hsp104*Δ diploid with a plasmid expressing either wild type *HSP104* or the *hsp104* M-domain mutants from the *HSP104* promoter. Heterozygous *HSP104*/*hsp104*Δ diploids maintain both strong and weak [*PSI*+] variants with no noticeable defect in propagation due to potential haploinsufficiency (data not shown). We first noticed that *hsp104-D434A* had a dominant curing effect and resulted in red [*psi*−] diploids (Figure 6A). Next, we sporulated the diploids, selected *hsp104*Δ haploids harboring the wild type or mutant Hsp104 plasmid, and then assessed [*PSI+*] propagation phenotypically. First, by the [*PSI*+]-dependent colorimetric assay, *hsp104-V426I* colonies appeared to sector, as observed originally. Note, however, that colonies grown on minimal media to select for the plasmid do not show as striking color development as they do on rich media. By contrast, cells expressing *hsp104-V426C*, *hsp104-D434A*, *hsp104-K480C*, or *hsp104-Y507D* appeared darker pink to red, similar to the vector control, thus indicating an impaired ability to propagate [*PSI*+] (Figure 6A). To determine whether these cells are propagating [*PSI*+] at all or are harboring any form of Sup35 aggregates, we performed semi-denaturing detergent agarose gel electrophoresis (SDD-AGE) with the haploids. We found that *hsp104-V426I*, *hsp104-V426C*, and *hsp104-K480C* cells still maintained aggregates of Sup35, while *hsp104-D434A* cells did not (Figure 6B). However, the distribution of Sup35 aggregates in *hsp104-V426C* and *hsp104-K480C* cells was shifted to a higher molecular weight as compared to wild type *HSP104* strong [*PSI*+] cells. This explains the weaker nonsense suppression phenotype [19], and suggests that these mutants are defective in fragmenting Sup35 aggregates. Unfortunately, for unknown reasons, SDD-AGE does not reliably show monomeric protein.

We next tested whether any of the mutants were capable of propagating a structurally distinct Sup35 aggregate species, a weak [*PSI*+] variant. Using the same approach as for strong [*PSI*+], we transformed weak [*PSI*+] heterozygous *HSP104*/*hsp104*Δ diploids with plasmids expressing either wild type *HSP104* or the M-domain mutants from the *HSP104* promoter. Similar to our observations with the strong [*PSI*+] diploid, *hsp104-D434A* dominantly cured diploids propagating a weak [*PSI*+] variant (Figure 6C). Since *hsp104-D434A* dominantly cures two distinct variants of [*PSI*+], this suggests that this mutation inhibits wild type Hsp104 function in mixed hexamers. Diploids harboring *hsp104-K480C* also appeared to have decreased nonsense suppression, suggesting that *hsp104-K480C* might also have a dominant curing effect on weak [*PSI*+] (Figure 6C). Next, we sporulated the diploids and isolated *hsp104*Δ haploids expressing the wild type or mutant Hsp104 to assess the color phenotype and the presence of Sup35 aggregates using SDD-AGE (Figure 6C,D). In contrast to strong [*PSI*+], we found that the only mutant able to propagate the weak variant of [*PSI*+] was Hsp104-V426C. This shows that these mutants differentially affect propagation of [*PSI*+] variants.

Interestingly, despite several attempts to generate strong or weak [*PSI*+] haploids expressing *hsp104-Y507D*, we were only able to isolate single haploids expressing *hsp104-Y507D* from both the strong and weak [*PSI*+] heterozygous diploids (Figure 6A,C). In fact, these haploids were unable to grow beyond the initial isolation and spotting (Figure 6A,C), and thus were not used in further biochemical analysis. In addition to sporulating diploids, we also attempted to replace wild type *HSP104* in a strong [*PSI*+] *hsp104*Δ strain with *hsp104-Y507D* by co-expressing both wild type *HSP104* and *hsp104-Y507D* and then eliminating the wild type *HSP104* plasmid. This method also proved unsuccessful in our attempts to isolate [*PSI*+] cells expressing Hsp104-Y507D. From these data, we propose that *hsp104-Y507D* is highly toxic in the presence [*PSI*+]. Indeed, expression of Hsp104-Y507D in [*psi*−] *hsp104*Δ cells did not show similar toxicity, suggesting that toxicity is dependent on Sup35 aggregation. Similar toxicity in the presence of [*PSI*+] has been observed for another M-domain mutant, *hsp104-A503V* [64], suggesting that prion-dependent toxicity is not specific for this one residue, but may be caused by a particular dysregulation of the M-domain.

### M-domain mutants are able to propagate distinct variants of [*RNQ*+]

We next examined the ability of the M-domain mutants to propagate several different variants of the [*RNQ*+] prion. Similar to [*PSI*+], the [*RNQ*+] prion is also sensitive to changes in Hsp104 activity and we previously showed that variants of [*RNQ*+] are differentially affected by changes in Hsp104 activity [30], [59], [65]. Variants of [*RNQ*+] have been characterized by their ability to induce the [*PSI*+] prion and by the Rnq1 aggregate pattern observed in cells by fluorescence [58], [66], [67]. [*RNQ*+] variants typically display either a single-dot (s.d.) or multiple-dot (m.d.) pattern of fluorescence that describes the appearance of Rnq1-GFP aggregates in [*RNQ*+] cells [66]. [*RNQ*+] variants that harbor the s.d. fluorescence pattern can facilitate the induction of [*PSI*+] at low, medium, high, and very high levels upon Sup35 over-expression. Only one established m.d. variant of [*RNQ*+] has been characterized and it exhibits a high rate of [*PSI*+] induction. We utilized these five [*RNQ*+] variants to ascertain the effect of the M-domain mutants on conformational variants of [*RNQ*+].

**Figure 7:**
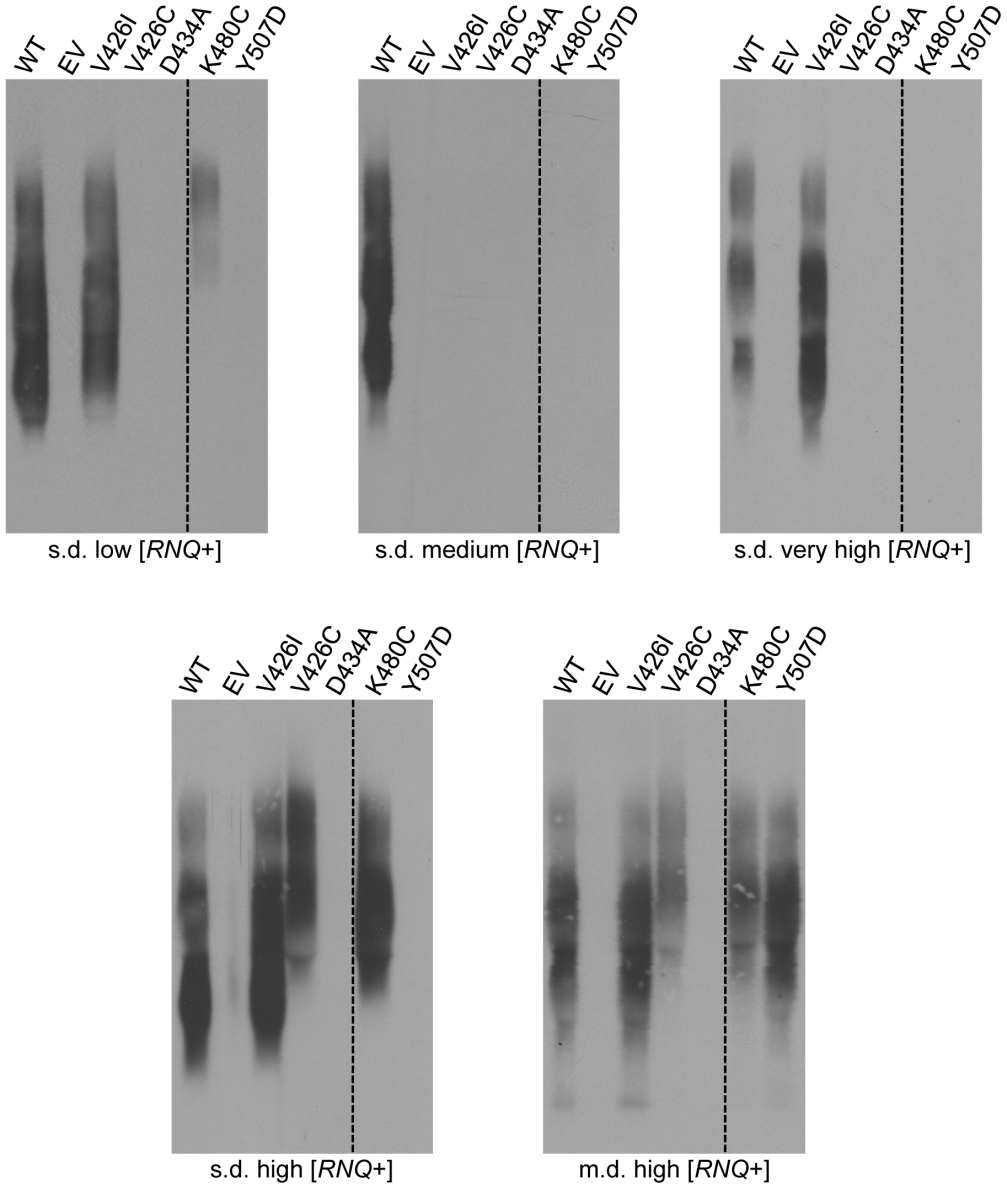
M-domain mutants differentially propagate [*RNQ*+] variants. *hsp104*Δ strains propagating the [*RNQ*+] variants, s.d. low, s.d. medium, s.d. high, s.d. very high, or m.d. high, and expressing *HSP104* (WT), *hsp104-V426I*, *hsp104-V426C*, *hsp104-D434A*, *hsp104-K480C*, *hsp104-Y507D*, or an empty vector control (EV) were subjected to SDD-AGE and western blot with an antibody against Rnq1. Dashed lines represent different parts of the same gel that have been cropped for clarity. Each SDD-AGE is one representative of at least three independent experiments.

We used *hsp104*Δ cells that were complemented by wild type *HSP104* from a plasmid and propagated one of the [*RNQ*+] variants as a starting point to replace *HSP104* with the M-domain mutants. We transformed the plasmids containing the Hsp104 M-domain mutants into these strains and subsequently selected for cells that eliminated wild type *HSP104* by growing them on media that counter-selected against cells containing the *URA3*-marked *HSP104* plasmid. Interestingly, we observed a differential effect of the mutants on the propagation of the [*RNQ*+] variants by both SDD-AGE analysis (Figure 7) and well-trap assay (data not shown). Of the M-domain mutants, *hsp104-V426I* cells were able to maintain all of the [*RNQ*+] variants except s.d. medium [*RNQ*+] (Figure 7). In fact, none of the Hsp104 mutants were able to maintain propagation of the s.d. medium [*RNQ*+] variant, suggesting that this prion conformation is most sensitive to changes in the Hsp104 M-domain activity. On the other hand, cells expressing *hsp104-D434A* did not propagate any of the tested variants of [*RNQ*+], suggesting that this mutant is a general prion inhibitor. Cells expressing *hsp104-V426C* only propagate s.d. high and m.d. high [*RNQ*+], while *hsp104-K480C* cells propagate these two variants along with s.d. low [*RNQ*+]. However, a larger aggregate size was maintained in these cells, suggesting that propagation is still impaired. Interestingly, m.d. high [*RNQ*+] was the only prion variant that was maintained in *hsp104-Y507D* cells.

## Discussion

Here, we present an analysis of five Hsp104 M-domain mutants, which have differential effects on chaperone function and cell viability. Our data provide further support that positioning of the M-domain is crucial to regulating the function of Hsp100 chaperones. Initially, we identified Hsp104-V426I from a screen for factors that affected [*PSI*+] propagation. We observed that *hsp104-V426I* cells had defects in [*PSI*+] propagation that manifested as a sectoring [*PSI*+] phenotype. We have reported this phenotype previously with other Hsp104 mutants that have varying effects on Hsp104 structure and function [22], but this was the only mutation we identified in the M-domain. The coiled-coil M-domain of Hsp104 is proposed to regulate ATPase activity, substrate disaggregation, and co-chaperone interactions [45]. We noted that the V426 residue in Hsp104 is analogous to the recently characterized L424 residue in ClpB, which plays a role in regulating the position and mobility of the M-domain in ClpB [48]. Previously, it was shown that the stability of the coiled-coil M-domain of ClpB depends on the leucine zipper-like interactions between leucine and isoleucine residues and that mutation of these residues to alanine caused significant changes in chaperone activity, ATP hydrolysis, and hexamer formation [68]. Perhaps, then, mutation of the valine at residue 426 to an isoleucine disrupts the normal isoleucine-leucine interactions, thereby resulting in slight destabilization of the M-domain. However, we do not have direct evidence that the V426I mutation affects the mobility of the M-domain of Hsp104. Yet, if this residue plays an analogous role to that of ClpB, then this residue contributes to M-domain positioning, and its mutation perturbs interactions of the M-domain with neighboring subunits within the hexamer.

In order to elucidate the effect of the V426I mutation on the function of the M-domain and activity of Hsp104, we examined the rates of ATP hydrolysis, hexamer formation, thermotolerance, and disaggregation. We also examined these same properties using a set of mutations in the Hsp104 M-domain. These mutations were analogous to mutations in ClpB that were proposed to stabilize either the repressed or de-repressed conformation of the M-domain, resulting in changes in the regulation of overall chaperone activity [48], [54]. Analyzing the homologous mutations in Hsp104, we found that, in general, the M-domain mutants had similar effects on the activity of Hsp104 as they displayed in ClpB, but some differences were noted (Table 1). (Importantly, the decreased steady state levels of Hsp104-V426C in yeast (Figure S1) do not explain our observed results.)

**Table 1:** Summary of biochemical and biological effects of the Hsp104 M-domain mutants.

	WT	V426I	V426C	D434A	K480C	Y507D
**ATPase Activity**	+	+	−	−	++	++
**Hexamerization**	+	+	−	−	+	+
**Temp. Sensitivity**	+	+	+	+	++	++
**Thermotolerance**	+	+	+	−	+/−	+/−
**Luciferase Refolding**	+	+/−	+/−	−	+/−	+/−
**Strong [** ***PSI*** **+] 2n**	+	+/−	+/−	−	+/−	+/−
**Weak [** ***PSI*** **+] 2n**	+	+	+/−	−	+/−	+/−
**Strong [** ***PSI*** **+] n**	+	+/−	+/−	−	+/−	NT
**Weak [** ***PSI*** **+] n**	+	−	+/−	−	−	NT
**s.d. low [** ***RNQ*** **+]**	+	+	−	−	+/−	−
**s.d. med [** ***RNQ*** **+]**	+	−	−	−	−	−
**s.d. high [** ***RNQ*** **+]**	+	+	+/−	−	+/−	−
**s.d. very high [** ***RNQ*** **+]**	+	+	−	−	−	−
**m.d. high [** ***RNQ*** **+]**	+	+	+/−	−	+/−	+

Effects of Hsp104 mutations were characterized as follows for the indicated properties and prion propagation, as compared to wild type (WT) Hsp104: (+) comparable to WT, (+/−) some defect, (−) abolished/cured, or (++) enhanced activity or sensitivity. NT: not tested, 2n: yeast diploids, n: yeast haploids.

The Hsp104-D434A mutation decreased ATPase activity and disaggregation activity, thereby supporting the prediction that this mutation stabilizes a repressed conformation of the M-domain and thereby inhibits overall Hsp104 activity [48]. The M-domain mutants Hsp104-K480C and Hsp104-Y507D, on the other hand, increased the rate of ATP hydrolysis and caused toxicity when expressed in cells grown at higher temperatures, indicative of a de-repressed conformation of the M-domain [48], [69]. Interestingly, our biochemical characterization suggested that both Hsp104-V426I and Hsp104-V426C did not stabilize either the repressed or the de-repressed conformation. Instead, we propose that the Hsp104-V426I and Hsp104-V426C mutations have moderate effects on the mobility and regulatory function of the M-domain. Moreover, the difference in the ATP hydrolysis rates of Hsp104-V426I and Hsp104-V426C suggest that the biochemical properties associated with the side chain of this residue are important [43]. Thus, the M-domain is finely tuned to regulate various functions of Hsp104 and disruption of this balance can lead to severe consequences for Hsp104 function.

Although several studies have examined the role of the M-domain in regulating protein disaggregation and ATPase activity [46], [48], [49], [52], [53], [63], much less is known about the effect of the Hsp104 M-domain regulatory function on yeast prion propagation. Here, we show that mutations that disrupt M-domain function also inhibit prion propagation. The repressed mutant Hsp104-D434A dominantly cured both strong and weak [*PSI*+] variants. Interestingly, the de-repressed mutants Hsp104-K480C and Hsp104-Y507D appear to have distinct effects on [*PSI*+] propagation despite having similar biochemical properties. While Hsp104-Y507D appears to be toxic in the presence of both strong and weak [*PSI*+], Hsp104-K480C is able to propagate strong [*PSI*+], but has an incomplete dominant inhibitory effect on weak [*PSI*+]. These data correlate well with observations that over-expression of Hsp104 cures weak [*PSI*+] variants more efficiently than strong [*PSI*+] variants [8]. One hypothesis to explain the observed differences between weak and strong [*PSI*+] is that weak [*PSI*+] variants are more dependent on Hsp70s and Hsp40s for efficient propagation, as varying levels of Hsp70 or Hsp40 expression can have greater effects on weak [*PSI*+] variants than strong variants [70], [71]. Indeed, Hsp104 acts in concert with Hsp70s and Hsp40s and the stoichiometric balance of this complex is an important variable in regulating protein disaggregation [1], [72], [73]. In fact, expression of ClpB in yeast is capable of prion propagation if it contains the M-domain of Hsp104 to maintain proper interactions with yeast co-chaperones, or if the yeast express the bacterial Hsp70 and its partner nucleotide exchange factor [63]. Furthermore, the de-repressed M-domain mutants of ClpB were shown to have reduced interaction with the KJE chaperones [54]. Therefore, perhaps a reduced interaction of Hsp104-K480C with co-chaperones is responsible for specifically curing the weak [*PSI*+] variant.

Similar to Hsp104-K480C, Hsp104-V426I and Hsp104-V426C differentially affect propagation of the [*PSI*+] variants. These mutations maintain strong [*PSI*+], albeit inefficiently, but either cure or alter the propagation of weak [*PSI*+]. It was previously demonstrated both in vitro and in vivo that Hsp104 has a decreased interaction with Sup35 structures that produce weak [*PSI*+], as compared to those that produce strong [*PSI*+] [20], [36]. In addition, we have recently found that decreased Hsp104 activity is sufficient to propagate strong but not weak variants of [*PSI*+] [22]. Therefore, the data we present in this study provide additional insight by showing that changes in the regulatory function of the M-domain is one mechanism that can alter the ability of Hsp104 to stably propagate distinct [*PSI*+] variants.

In addition to changes in [*PSI*+] propagation, we also found differential effects of the M-domain mutants on the propagation of conformational variants of the [*RNQ*+] prion. The repressed M-domain Hsp104-D434A mutant cannot propagate any tested variant of [*RNQ*+]. As we have previously characterized mutants of Hsp104 that display decreased activity, but are still able to propagate specific variants of [*RNQ*+] [22], [59], there is clearly a threshold of activity that exists that is required for [*RNQ*+] propagation. Our data suggest that the activity of Hsp104-D434A does not meet this threshold. Interestingly, none of the M-domain mutants were able to propagate s.d. medium [*RNQ*+], and Hsp104-Y507D maintained propagation of only the m.d. high [*RNQ*+] variant. Besides modulating interactions with co-chaperones, another hypothesis for such differential prion variant propagation is that the stability of the prion variant dictates the requirement for Hsp104 activity in prion maintenance [20]. Indeed, the decreased stability of m.d. high [*RNQ*+] [74] may help explain why this prion conformer can still propagate in *hsp104-Y507D* cells, while the other [*RNQ*+] variants cannot. However, the s.d. [*RNQ*+] variants have been shown to have similar stabilities [74], yet are differentially propagated by the Hsp104 M-domain mutants. This suggests that aggregate stability is only one contributing factor to Hsp104 dependency, and that the ability of co-chaperones to interact with prion aggregates and Hsp104 likely plays an additional major role in dictating the propagation of different prion variants. Therefore, our data clearly demonstrate the complexity of prion variant propagation and illustrate the need for further investigation to understand the mechanism of interaction between chaperones and conformationally distinct prion variants.

The M-domain clearly plays a crucial role in regulating Hsp104/ClpB function. However, the structure and function of the Hsp104/ClpB M-domain has been a subject of much investigation and controversy in recent years. Various structural studies of ClpB and Hsp104 have proposed significantly different models for the position of the M-domain in relation to the hexameric structure [55], [56], [75]. Specific residues in the M-domain are protected, suggesting that at least part of the M-domain is tightly packed into or against the body of the hexamer [48], [52], [56]. Additionally, cross-linking and fluorescence quenching experiments suggest that the M-domain contacts residues in the NBD1, either in the neighboring subunit or in the same subunit [48]. The flexibility of the M-domain to break and re-form these contacts is integral to the regulation of chaperone function [43], [48], [54]. While the data in our study do not lend direct support to any one structural model, our data show that the M-domain of Hsp104 plays a key role in regulating the disaggregation of both prion and non-prion substrates. This supports the findings from several other studies that show that mutations in the coiled-coil M-domain affect all of the distinct activities that Hsp104/ClpB possesses [42], [43], [47], [48], [49], [52], [54], [64]. This suggests that this domain may be the master regulator of Hsp104/ClpB function. As such, elucidation of the regulatory mechanism of the M-domain is vital to understanding the disaggregation mechanism of Hsp104/ClpB.

## Supporting Information

Figure S1 **Expression of Hsp104 mutants.** *hsp104*Δ cells harboring plasmids expressing *HSP104* (WT), *hsp104-V426I*, *hsp104-V426C*, *hsp104-D434A*, *hsp104-K480C*, or *hsp104-Y507D* from the native *HSP104* promoter, or an empty vector control (EV), were grown at 30°C to an OD_600_ ∼1.0, lysed, and subjected to SDS-PAGE and western blot using anti-Hsp104 and anti-Pgk1 antibodies.
(TIF)
