# Supplementary figures and images for: Regulation of the Hsp104 Middle Domain Activity Is Critical for Yeast Prion Propagation
Source: PLoS One. 2014 Jan 23;9(1):e87521. doi: 10.1371/journal.pone.0087521 (PMC3900729; doi:10.1371/journal.pone.0087521)

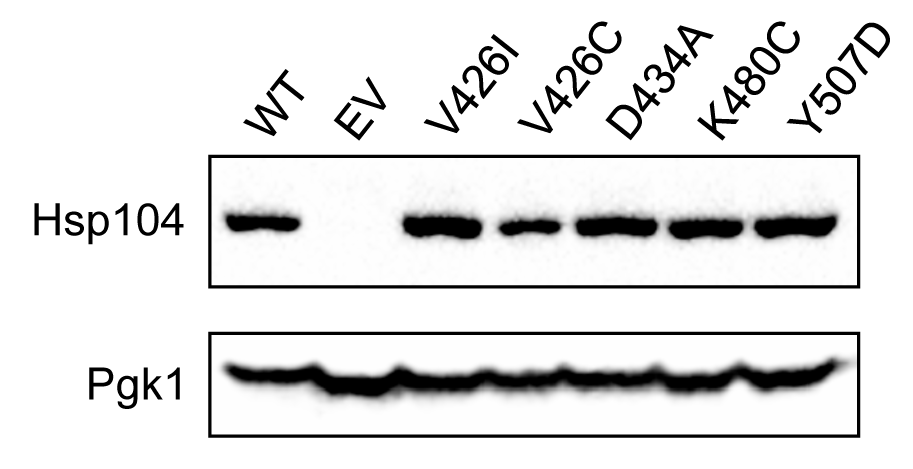

Supplement: Figure S1 — Expression of Hsp104 mutants. hsp104Δ cells harboring plasmids expressing HSP104 (WT), hsp104-V426I, hsp104-V426C, hsp104-D434A, hsp104-K480C, or hsp104-Y507D from the native HSP104 promoter, or an empty vector control (EV), were grown at 30°C to an OD600 ∼1.0, lysed, and subjected to SDS-PAGE and western blot using anti-Hsp104 and anti-Pgk1 antibodies. (TIF) [file pone.0087521.s001.tif]
